# The Real-World Results of the Single Intravitreal Injection of Faricimab in Treatment-Naïve Subfoveal Myopic Choroidal Neovascularization

**DOI:** 10.3390/medicina62050832

**Published:** 2026-04-27

**Authors:** Hao-Chun Chang, Ling-Uei Wang, Tzu-Lun Huang, Pei-Yao Chang, Wei-Ting Ho, Yung-Ray Hsu, Fang-Ting Chen, Yun-Ju Chen, Cheng-Hung Lin, Jia-Kang Wang

**Affiliations:** 1 Department of Ophthalmology, Far Eastern Memorial Hospital, New Taipei City 220, Taiwan; 2 Department of Medicine, National Yang Ming Chiao Tung University, Taipei City 112, Taiwan; 3 Department of Electrical Engineering, Yuan Ze University, Taoyuan City 320, Taiwan; 4 Department of Medicine, National Taiwan University, Taipei City 100, Taiwan; 5 Department of Electrical Engineering, National Taiwan Normal University, Taipei City 106, Taiwan

**Keywords:** intravitreal injection, faricimab, myopic choroidal neovascularization

## Abstract

*Background and Objectives*: Myopic choroidal neovascularization (mCNV) is a vision-threatening complication of pathologic myopia. While anti-VEGF monotherapy is the current standard of care, recurrence and suboptimal responses remain challenges. Faricimab is a novel bispecific antibody that targets both vascular endothelial growth factor (VEGF) and angiopoietin-2 (Ang-2) to improve vascular stability. This study aims to evaluate the short-term efficacy and safety of a single intravitreal faricimab injection in eyes with active mCNV. *Materials and Methods*: This retrospective, single-center study included 27 eyes from 24 patients with active mCNV, including both treatment-naïve and previously treated cases. All eyes received a single intravitreal injection of faricimab (6.0 mg/0.05 mL). Best-corrected visual acuity (BCVA) in logMAR and central retinal thickness (CRT) via spectral-domain optical coherence tomography were assessed at baseline and one month post injection. Statistical significance was determined using paired and independent *t*-tests (*p* < 0.05). *Results*: The study population (mean age 55.5 ± 13.9 years; mean axial length 29.3 ± 1.6 mm) showed significant improvements at one month. Mean BCVA improved from 0.77 ± 0.71 logMAR to 0.51 ± 0.52 logMAR (*p* < 0.005). Mean CRT decreased from 290.2 ± 66.0 μm to 242.5 ± 45.7 μm (*p* < 0.005). No ocular adverse events, such as intraocular inflammation, retinal detachment, or endophthalmitis, were observed. *Conclusions*: A single intravitreal injection of faricimab provides significant short-term functional and anatomical improvement in this small retrospective series. Dual inhibition of VEGF-A and Ang-2 appears to be a safe and effective approach for stabilizing retinal vasculature in patients with high myopia. Larger, long-term prospective studies are needed to determine optimal treatment intervals for mCNV.

## 1. Introduction

Myopic choroidal neovascularization (mCNV) is a significant cause of blindness among individuals with high myopia, particularly impacting those during their working years [1,2]. The prevalence of mCNV ranges from 5.2% to 11.3% among patients with pathologic myopia and 0.04% to 0.05% in the general population [3]. The global rise in myopia and high myopia, especially in East and Southeast Asia, highlights the importance of addressing mCNV due to its severe effects on productivity and quality of life [2,4,5]. Without treatment, mCNV can lead to macular bleeding, edema, and scarring, resulting in poor visual prognosis and potential blindness [5]. Studies show that over 20% of patients are legally blind at diagnosis, increasing to 53–96% at follow-up [5]. A ten-year study reported that 88.9% of affected individuals developed severe visual impairment (visual acuity < 20/200) within five years [6,7].

Historically, treatment options for mCNV included laser photocoagulation, photodynamic therapy with verteporfin (vPDT), and macular surgery. These methods did not significantly improve visual outcomes or recurrence prevention compared to anti-vascular endothelial growth factor (anti-VEGF) therapy [8]. Vascular endothelial growth factor (VEGF) is a key factor in the pathogenesis of choroidal neovascularization (CNV), with elevated levels found in the aqueous humor of mCNV-affected eyes [9]. Currently, anti-VEGF therapy is the first-line treatment for mCNV, employing agents such as ranibizumab, aflibercept, and off-label bevacizumab [10]. A 2016 meta-analysis of randomized controlled trials revealed that anti-VEGF therapy significantly improved best-corrected visual acuity (BCVA) compared to vPDT, laser photocoagulation, and sham therapy [11]. While anti-VEGF monotherapy is effective, many patients require frequent injections and long-term monitoring to manage recurrences. This represents a significant treatment burden, particularly for working-age patients who face productivity losses due to frequent clinical visits.

The pathogenesis of mCNV involves a complex interplay of angiogenic factors and vascular instability. In pathologic myopia, the mechanical stretching of the retina creates a pro-angiogenic environment where VEGF levels are upregulated. While VEGF promotes vascular permeability and neovascular growth, angiopoietin-2 (Ang-2) acts as a context-dependent antagonist of Tie2 signaling. Elevated Ang-2 levels destabilize the vascular endothelium, increasing its sensitivity to VEGF and promoting inflammation and vascular leakage [12].

Faricimab is a novel agent designed to address these dual pathways, which allows the molecule to bind and neutralize both Ang-2 and VEGF-A simultaneously. This dual-pathway inhibition is unique compared to traditional monotherapies, as it aims not only to reduce neovascularization via anti-VEGF action but also to enhance vascular stability and reduce inflammation through Ang-2 inhibition. Faricimab has demonstrated optimal safety and treatment efficacy in treatment-naïve neovascular age-related macular degeneration (nAMD) in the phase III studies TENAYA and LUCERNE for 2 years [13]. The real-world study TRUCKEE demonstrated satisfying efficacy and safety up to six months after faricimab injection in treatment-naïve as well as previously treated patients with nAMD [14]. Another study in Japan investigated treatment-naïve nAMD and polypoidal choroidal vasculopathy (PCV) patients receiving three consecutive monthly intravitreal injections of faricimab, revealing functional and anatomical improvements [15].

However, there is neither a clinical trial nor real-world study regarding faricimab in treatment-naïve mCNV patients. This study examines the off-label use of faricimab for mCNV management, providing insights into its short-term efficacy and associated adverse events.

## 2. Materials and Methods

### 2.1. Study Design

This investigation was designed as a retrospective cohort study. We reviewed the medical records of all patients with active treatment-naïve mCNV who received faricimab between April 2022 and October 2024. This study adhered to the principles outlined in the Declaration of Helsinki and received approval from the Institutional Review Board of Far Eastern Memorial Hospital in Taiwan (IRB number 113249-E). Informed consent was waived because of the study’s retrospective nature and the fact that the analysis used anonymous clinical data. The clinical decision for the off-label use of faricimab was made in the best interest of the patients, following institutional protocols for off-label medications and a thorough discussion of the potential benefits and available standard anti-VEGF alternatives. The faricimab used in this study was provided as free samples; therefore, no additional drug costs were incurred by the patients for this off-label treatment.

Eligibility inclusion criteria required aged over 18 years, highly myopic with axial length exceeding 26 mm, or myopia higher than −6 diopters in phakic eye without obvious cataract/prior laser vision correction/radial keratotomy. The patients’ diagnosis was confirmed with the presence of subfoveal choroidal neovascularization on spectral-domain optical coherence tomography (SD-OCT, AngioVue, Optovue Inc., San Francisco, CA, USA), and the existence of submacular leakage on the fluorescein angiography (TRC-NW7SF, Topcon Inc., Tokyo, Japan) [16].

Exclusion criteria comprised the presence of other retinal diseases, such as PCV, retinal vein or artery occlusion, diabetic retinopathy, angioid streaks, and vitelliform macular dystrophy, pregnancy or nursing status, a history of thromboembolic events, intraocular surgery including pars plana vitrectomy and cataract surgery within the preceding three months or planned within the next 28 days, uncontrolled hypertension, known coagulation abnormalities, current use of anticoagulant medications other than aspirin, or the presence of iris neovascularization/vitreous hemorrhage.

All participants received single intravitreal injections of faricimab 6 mg in 0.05 mL (Vabysmo, F. Hoffmann-La Roche Ltd., Kaiseraugst, Switzerland). To ensure reproducibility, all procedures were performed in a dedicated sterile clean operation room under strict aseptic conditions. The periocular skin and conjunctival sac were disinfected using 5% povidone-iodine (diluted from 10% povidone-iodine, Saint-Iodine; Taiwan Patron chemical & pharmaceutical Co., Kaohsiung, Taiwan) after topical anesthesia (0.5% proparacaine hydrochloride; Alcon, Fort Worth, TX, USA) was administered. A Lancaster eyelid speculum was utilized to maintain exposure and to cover eyelashes. The injection was administered 3.5 to 4.0 mm posterior to the limbus through the pars plana using a 30-gauge needle.

Patients were followed 1 week after the injection for adverse-event monitoring, including intraocular pressure (IOP) and anterior/posterior segment examination via slit-lamp biomicroscope. Examinations of BCVA with Snellen charts (converted to logMAR and ETDRS letters for statistical analysis), anterior/posterior segment examination, and SD-OCT at 1 month were recorded. These naïve patients received only first single faricimab for mCNV treatment. Because the patients underwent treatment with other anti-VEGF agents after reimbursement from National Health Insurance on the next month or at the time of recurrence, we collected short-term data with one month following single injection of faricimab for these highly myopic patients.

### 2.2. Outcome Measures

Primary outcome measures included changes in BCVA, central foveal thickness (CFT) in the central 1 mm as measured by OCT, and dry macula rate (defined as no intraretinal or subretinal fluid on SD-OCT) from baseline to month one. Injection-related complications were also recorded. Statistics were performed using Prism 10, a software powered by GraphPad software company, Boston, MA, USA. The CFT and BCVA changes of pre- and post-injection data for each eye were compared using the Wilcoxon signed-rank test. The significance threshold is set at *p* < 0.05.

## 3. Results

### 3.1. Demographics

Following the application of eligibility criteria, a total of 27 eyes from 24 patients were included in the final analysis. The baseline characteristics are presented in Table 1. The study population had a mean age of 55.5 ± 13.9 years and was predominantly female (19/24). The mean axial length was 29.3 ± 1.6 mm. At baseline, patients exhibited poor visual acuity (mean BCVA 0.77 ± 0.71 logMAR) and increased foveal thickness (mean CFT 290.2 ± 66.0 μm).

### 3.2. Outcomes

**Figure 1:**
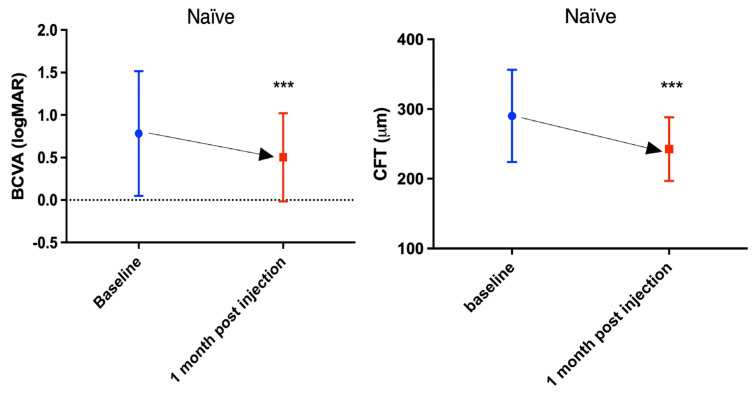
Best-corrected visual acuity (BCVA) (left) and central foveal thickness (CFT) (right) improvement one month following single injection of faricimab, *** *p* < 0.005.

**Figure 2:**
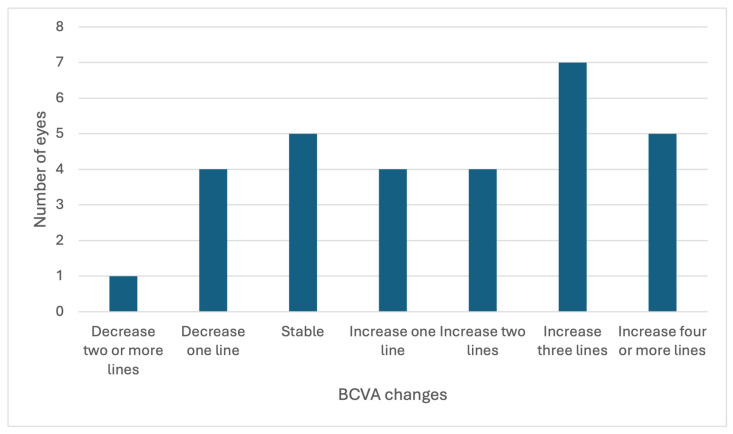
Distribution of best-corrected visual acuity (BCVA) changes one month following single faricimab injection.

**Figure 3:**
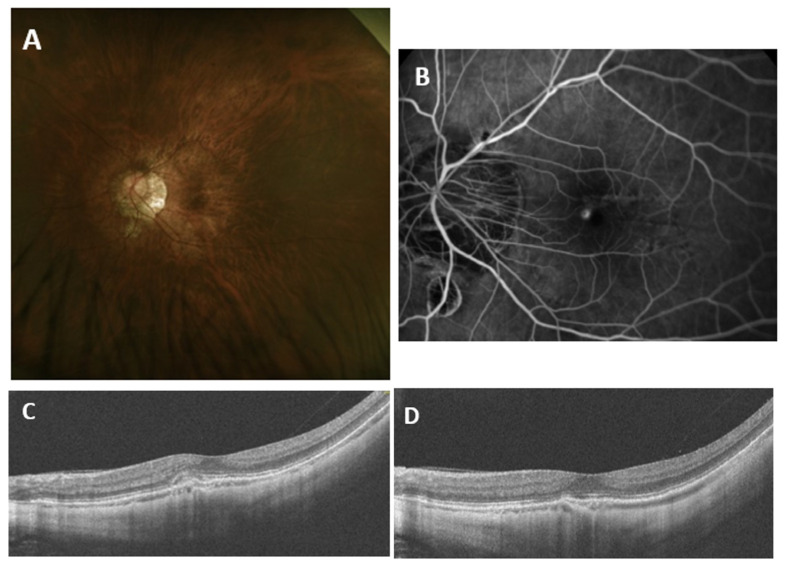
Multimodal images in the left eye of one treatment-naïve case. (**A**): Color fundus photography showing macular lesion; (**B**): fundus fluorescein angiography showing macular leakage; (**C**): myopic choroidal neovascularization demonstrated on optical coherence tomography with subretinal fluid; (**D**): subretinal fluid disappeared one month following single faricimab injection.

Upon 1 month follow-up after the single intravitreal injection of faricimab, BCVA increased and CFT reduced significantly (*p* < 0.005) (Table 2, Figure 1). The average improvement of BCVA was 2.5 lines. About one-fourth of the patients experienced at least three lines of BCVA gains. One of the patients experienced more than one line of BCVA loss. (Figure 2). Approximately 80% of the patients achieved dry macula one month after single faricimab injection. For those eyes without dry macula, decreased subretinal or intraretinal fluid were observed. No adverse event was observed in all the patients. A representative case of treatment response is shown in Figure 3.

## 4. Discussion

This study represents the first real-world investigation of faricimab in treatment-naïve patients with mCNV. Faricimab was associated with improvements in both anatomical and functional outcomes. One month after a single intravitreal dose of faricimab, improvements in visual acuity and macular thickness were observed.

### 4.1. Functional Changes

Treatment-naïve patients with mCNV treated with ranibizumab in the RADIANCE study achieved a visual gain of about 8–9 Early Treatment Diabetic Retinopathy Study (ETDRS) letters after first intravitreal injection. The visual improvement progressed to +10.5–+10.6 letters following three months of treatment with 1.8 to 2.5 injections [17]. In the MYRROR study, eyes with naïve mCNV treated with aflibercept achieved a BCVA increase of approximately 7–8 letters after first aflibercept treatment. Improvement of BCVA up to 12.1 letters at 6 months with mean 2.6 injections was observed [17,18]. The MYRROR study also reported that nearly 15% of the patients achieved visual gains of more than 15 letters after first aflibercept injection, and 32.2% after 3-month follow-up. In comparison, among the 27 treatment-naïve eyes following single faricimab injection, the average improvement in BCVA was −0.26 logMAR, corresponding to an adjusted mean gain of +10.9 letters. In these patients, 33% experienced at least three lines of BCVA gains, equally to 15-letter improvement.

The current real-world study using faricimab treatment for mCNV achieved comparable visual improvements to aflibercept and ranibizumab in prior randomized controlled trials. Faricimab demonstrated similar short-term improvements of approximately +10-letter visual gains within 1 month. In contrast, prior studies using other anti-VEGF agent treatments required 3 to 6 months with several injections to achieve similar visual improvement. Our findings provide exploratory real-world data suggesting a rapid initial response. However, these observations are strictly preliminary and cannot be used to establish comparable or superior efficacy due to the non-comparative study design, and randomized controlled trials are warranted to provide more robust comparisons and to confirm whether faricimab offers superior early efficacy over established monotherapies.

Another common disease associated with CNV was nAMD. The phase III TENAYA and LUCERNE trials showed achievement of rapid visual improvement of nAMD with faricimab. Approximately 5-letter gains after first faricimab intervention were noticed, comparing to 6.2-letter improvement after 12-month treatment for nAMD [14,15]. The real-world TRUCKEE study also showed BCVA improvements of 4.9 letters in treatment-naïve cases with nAMD after initial single faricimab injection. More visual gains of 8.1 letters were accomplished following 3 monthly injections of faricimab for the patients [15]. Theses study results indicate faricimab can similarly reverse the pathogenesis of CNV in patients with high myopia following the first injection. Consecutive or continuous faricimab treatment can probably further improve the visual outcome for mCNV.

### 4.2. Anatomical Changes

In the RADIANCE study, ranibizumab achieved a significant mean macular thickness reduction, ranging from −61.0 to −77.6 μm at 3 months [17]. In the MYRROR study, patients treated with aflibercept achieved an average macular thickness decrease of −80.7 μm at 6 months, with the most significant reduction occurring after the first two injections in the initial 8 weeks [19]. Among our patients, mean CFT decreased 47.7 μm after single faricimab injection. Although ranibizumab and aflibercept demonstrated greater macular thinning, faricimab achieved a significant and clinically meaningful reduction within 1 month, underscoring its rapid therapeutic efficacy. The TENAYA and LUCERNE trials revealed rapid anatomical improvement of faricimab for nAMD. During the initial three continuous monthly injections, mean central subfield thickness reduced approximately 130 μm after the first injection, with further decrease to 145.4 μm after three injections. In addition, faricimab attained significantly higher macular thickness reduction comparing to aflibercept at visits in the first 3 months [20]. The results infer further decrease in CFT may occur following more faricimab treatment for mCNV, and more rapid macular thickness reduction than other anti-VEGF drugs.

The RADIANCE study reported that approximately 64–66% of patients achieved CNV leakage resolution at 3 months [17]. The MYRROR study reported that 86.4% of patients had no CNV leakage by 6 months, with most improvements occurring in the first 8 weeks [19]. Dry macula was accomplished in 81% of our patients after one-month management. These findings suggest that faricimab achieves comparable or even higher ability of fluid eradication in a short timeframe than ranibizumab and aflibercept for eyes with mCNV. The post hoc analysis of the TENAYA and LUCERNE studies demonstrated absence of subretinal and intraretinal fluid can be attained with significantly greater possibility in 60% of patients after first faricimab injections than in 49% of those following the first aflibercept intervention [20]. It was also revealed that 77% of patients after three initially continuous faricimab injections achieved dry macula, and more than 67% of those following three aflibercept injections. The outcomes indicate that more patients perhaps have dry macula after additional faricimab therapy for mCNV, and higher dry macula rate than other anti-VEGF drugs.

Improvement of BCVA and CFT along with dry macula rates in most patients with mCNV within 1 month of faricimab treatment was observed. These results highlight the potential clinical advantage of faricimab in providing rapid and robust efficacy for managing mCNV. Given that many mCNV patients are of working age, the rapid response observed might allow for extended treatment intervals earlier in the disease course, reducing the frequency of clinic visits and the associated socio-economic burden. In addition, additional vascular stabilizing action of anti-Ang-2 may explain some aspects of faricimab’s superiority to aflibercept for treatment of nAMD or diabetic macular edema [21].

The safety profile of faricimab was excellent in our study. No major ocular or systemic adverse events were reported, such as endophthalmitis, retinal detachment, intraocular inflammation, retinal vasculitis, retinal vascular occlusion, and thromboembolic disorders. This aligns with the favorable outcomes observed in the TENAYA and LUCERNE trials, which reported a safety profile comparable to aflibercept. In the TRUCKEE study, among 94 cases, there was one case each of intraocular inflammation and infectious endophthalmitis, which resolved with appropriate treatment. The Japanese study, among 62 cases, reported two cases of retinal pigment epithelium tears but no systemic complications. Overall, the current study highlights rapid and robust therapeutic effects of faricimab in improving BCVA, reducing CFT, and achieving high dry macula rates within a short timeframe. These findings establish its clinical advantage in managing mCNV and other neovascular diseases such as nAMD and PCV.

### 4.3. Limitations

Despite the promising results of this study, several limitations should be acknowledged. The relatively small sample size and short follow-up period may restrict the generalizability and long-term applicability of the findings. The retrospective study design and lack of randomization also introduce potential biases and limit the ability to establish causality between faricimab treatment and observed outcomes. Future research should include a larger sample size and incorporate randomized study designs to validate these findings further. Long-term follow-up is also essential to assess the therapeutic durability of faricimab and its safety profile over extended periods. Such studies should evaluate a “Treat-and-Extend” protocol to determine if faricimab’s dual-pathway inhibition can safely extend injection intervals beyond current standards, thereby further reducing the socio-economic and treatment burden for working-age patients. Furthermore, distribution of visual changes after faricimab treatment was variable in the research. Associated factors of visual prognosis should be analyzed. For instance, better visual improvement was relevant to younger age, poorer baseline BCVA, shorter axial length, and higher central retinal thickness following aflibercept injections for mCNV described in the MYRROR study [19]. But the issue regarding the visual outcomes cannot be investigated owing to small sample size. Finally, a more granular analysis of baseline predictive factors—including age, axial length, and initial central foveal thickness—is required to identify specific patient subgroups that may derive the greatest benefit from early faricimab intervention.

## 5. Conclusions

In conclusion, a single intravitreal injection of faricimab was associated with significant short-term functional and anatomical improvements in this small retrospective series. No safety concerns were identified in this small cohort. While these exploratory results are promising, large-scale prospective randomized controlled trials are necessary to determine long-term efficacy and compare faricimab directly with established anti-VEGF therapies for mCNV.

## Figures and Tables

**Table 1:** Patient demographics.

	Naïve (*n* = 27)
Age (years)	55.5 ± 13.9
Gender (male:female)	5:19
Lens status (phakic, without obvious cataract/LVC/RK:pseudophakic)	16:11
Axial length (mm) ^a^	29.3 ± 1.6
Mean spherical equivalent of phakic patient (D)	−11.53 ± 5.2
Central foveal thickness (μm)	290.2 ± 66.0
Best-corrected visual acuity (logMAR)	0.77 ± 0.71

^a^ Three patients were enrolled by spherical equivalent and had no records of axial length.

**Table 2:** Best-corrected visual acuity (BCVA) and central foveal thickness (CFT) changes and dry macula rate one month after single injection of faricimab (* *p* < 0.05).

	Naïve (*n* = 27)	
BCVA (logMAR)		*p*-value
Baseline	0.77 ± 0.71	0.0003 *
After injection	0.51 ± 0.52	
CFT (μm)		*p*-value
Baseline	290.2 ± 66.0	0.0002 *
After injection	242.5 ± 45.7	
Changes in BCVA (logMAR)	−0.26 ± 0.40	
Changes in CFT (μm)	−47.70 ± 71.07	
BCVA gains ≥ 3 lines	9/27 (33%)	
BCVA loss > 1 line	1/27 (3.7%)	
Dry macula rate	81% (22/27)	

## Data Availability

The data that support the findings of this study are available from the corresponding authors, J.-K.W. and L.-U.W., upon reasonable request.
